# Sphingomyelin and progression of renal and coronary heart disease in individuals with type 1 diabetes

**DOI:** 10.1007/s00125-020-05201-9

**Published:** 2020-06-20

**Authors:** Drazenka Pongrac Barlovic, Valma Harjutsalo, Niina Sandholm, Carol Forsblom, Per-Henrik Groop, on behalf of the FinnDiane Study Group

**Affiliations:** 1 University Medical Center Ljubljana, Ljubljana, Slovenia; 2 Faculty of Medicine, University of Ljubljana, Ljubljana, Slovenia; 3 Folkhälsan Institute of Genetics, Folkhälsan Research Center Biomedicum Helsinki, University of Helsinki, Haartmaninkatu 8, PO Box 63, FIN-00014 Helsinki, Finland; 4 Abdominal Center, Nephrology, University of Helsinki and Helsinki University Hospital, Helsinki, Finland; 5 Research Program for Clinical and Molecular Metabolism, Faculty of Medicine, University of Helsinki, Helsinki, Finland; 6 National Institute for Health and Welfare, Helsinki, Finland; 7 Department of Diabetes, Monash University, Melbourne, Victoria Australia

**Keywords:** Albuminuria, Coronary artery disease, Diabetic kidney disease, Glomerular filtration rate, Lipids, NMR metabolomics, Sphingomyelin

## Abstract

**Aims/hypothesis:**

Lipid abnormalities are associated with diabetic kidney disease and CHD, although their exact role has not yet been fully explained. Sphingomyelin, the predominant sphingolipid in humans, is crucial for intact glomerular and endothelial function. Therefore, the objective of our study was to investigate whether sphingomyelin impacts kidney disease and CHD progression in individuals with type 1 diabetes.

**Methods:**

Individuals (*n* = 1087) from the Finnish Diabetic Nephropathy (FinnDiane) prospective cohort study with serum sphingomyelin measured using a proton NMR metabolomics platform were included. Kidney disease progression was defined as change in eGFR or albuminuria stratum. Data on incident end-stage renal disease (ESRD) and CHD were retrieved from national registries. HRs from Cox regression models and regression coefficients from the logistic or linear regression analyses were reported per 1 SD increase in sphingomyelin level. In addition, receiver operating curves were used to assess whether sphingomyelin improves eGFR decline prediction compared with albuminuria.

**Results:**

During a median (IQR) 10.7 (6.4, 13.5) years of follow-up, sphingomyelin was independently associated with the fastest eGFR decline (lowest 25%; median [IQR] for eGFR change: <−4.4 [−6.8, −3.1] ml min^−1^ [1.73 m^−2^] year^−1^), even after adjustment for classical lipid variables such as HDL-cholesterol and triacylglycerols (OR [95% CI]: 1.36 [1.15, 1.61], *p* < 0.001). Similarly, sphingomyelin increased the risk of progression to ESRD (HR [95% CI]: 1.53 [1.19, 1.97], *p* = 0.001). Moreover, sphingomyelin increased the risk of CHD (HR [95% CI]: 1.24 [1.01, 1.52], *p* = 0.038). However, sphingomyelin did not perform better than albuminuria in the prediction of eGFR decline.

**Conclusions/interpretation:**

This study demonstrates for the first time in a prospective setting that sphingomyelin is associated with the fastest eGFR decline and progression to ESRD in type 1 diabetes. In addition, sphingomyelin is a risk factor for CHD. These data suggest that high sphingomyelin level, independently of classical lipid risk factors, may contribute not only to the initiation and progression of kidney disease but also to CHD.

**Figure:**
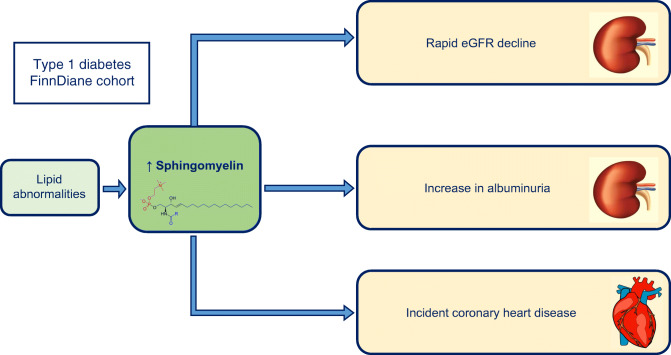
Graphical abstract

**Electronic supplementary material:**

The online version of this article (10.1007/s00125-020-05201-9) contains peer-reviewed but unedited supplementary material, which is available to authorised users.

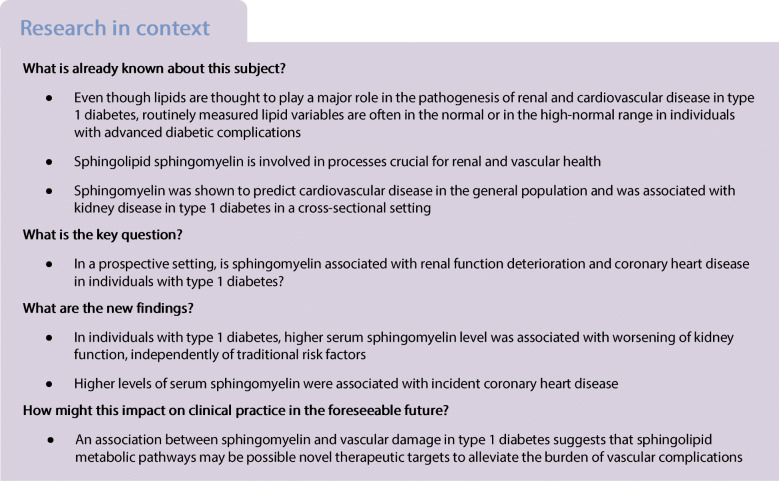



## Introduction

Diabetic kidney disease (DKD) is a fundamental risk factor for CVD in individuals with type 1 diabetes [1]. Notably, both DKD and CVD share common risk factors, such as glycaemic and blood pressure control. However, the link between these conditions remains ill-defined.

Among the CVD risk factors, the lipids have been highlighted as the most important determinant of CVD in diabetes [2], but lipids are also associated with the onset and progression of albuminuria in individuals with type 1 diabetes [3]. Although statin treatment targeting mainly LDL-cholesterol is a cornerstone of CVD prevention, it cannot correct for all the complex lipid disarrangements that operate in diabetes. Intriguingly, studies evaluating the statin effect on the renal disease progression have been inconclusive [4].

Sphingomyelin is a sphingolipid, involved in many processes crucial for renal and vascular health, including lipoprotein uptake, cell survival, proliferation, apoptosis, differentiation and immunological recognition [5]. Recently, preclinical studies have clearly shown that the sphingomyelin metabolic pathway is involved in podocyte injury [6] as well as atherosclerotic plaque inflammation [7]. Studies of sphingomyelin metabolism and DKD in individuals with type 1 diabetes are, however, rare and have yielded conflicting results [8, 9]. In addition, plasma sphingomyelin concentration was associated with incident CVD in the general population [10–12], whereas there is no similar study performed in individuals with type 1 diabetes yet.

In a metabolomic screen recently performed by our group, sphingomyelin was identified as the strongest contributor to kidney disease from the long list of lipid subclasses [8]. Furthermore, we were able to show that the urinary albumin excretion rate is directly linked to triacylglycerols and cholesterol-rich lipoproteins via sphingomyelin [13]. Therefore, in this study we aimed to investigate, in a prospective setting, whether sphingomyelin associates with kidney function deterioration and cardiovascular events independently of other traditional risk factors, in a large, well-characterised cohort of individuals with type 1 diabetes.

## Methods

### Study sample

This study is part of the ongoing Finnish Diabetic Nephropathy (FinnDiane) Study, which is a nationwide, prospective, multicentre study exploring the risk factors for diabetes complications, and diabetic nephropathy in particular. A detailed description of the protocol has previously been published [14]. In the present analysis we included individuals with an available sphingomyelin measurement at baseline (*n* = 1267). Individuals with inconclusive data on albuminuria status (*n* = 33) as well as without a follow-up visit (*n* = 147) were excluded from the analyses. Thus, a total of 1087 individuals were eligible for the analysis, and they all gave their written, informed consent to participate. The local ethical committees of the participating centres as well as the ethical committee of the Helsinki and Uusimaa Hospital District approved the study protocol, and the study was performed in accordance with the Declaration of Helsinki.

### Subject characteristics

#### Baseline evaluation

Type 1 diabetes mellitus was defined as the onset of diabetes before the age of 40 years and permanent insulin treatment initiated within 1 year of diagnosis. Baseline evaluation was performed from 1997 to 2001. At baseline, patient history and anthropometric data (weight, height, waist and hip circumferences) were collected by a trained nurse. Blood pressure was measured twice within 2 min intervals in the sitting position after a 10 min rest. Smoking was assessed using questionnaires. Data on sex were self-reported and all patients were white. Blood samples were collected after a light breakfast and analysed for HbA_1c_, creatinine and lipids. Serum sphingomyelin, together with other specific lipoprotein subclasses, was quantified by NMR spectroscopy. The protocols for the NMR experiment have been described elsewhere [13]. Severe diabetic retinopathy was defined based on a history of retinal laser treatment.

Renal status was assessed with both GFR and albuminuria. GFR was estimated by the Chronic Kidney Disease Epidemiology Collaboration (CKD-EPI) formula at baseline and during follow-up [15]. Classification of renal function was made using the Kidney Disease: Improving Global Outcomes (KDIGO) categories for eGFR. For individuals with end-stage renal disease (ESRD), eGFR was set at 10 ml min^−1^ 1.73 m^−2^ for the analyses that required a continuous eGFR value.

Urinary AER (UAER) was measured from 24 h [mg/24 h] or timed overnight [μg/min] urine collections. Normoalbuminuria was defined when, in at least two out of three urinary samples, UAER was <20 μg/min or <30 mg/24 h; microalbuminuria when UAER was ≥20 and <200 μg/min or ≥30 and <300 mg/24 h; or macroalbuminuria when UAER was ≥200 μg/min or ≥300 mg/24 h. In addition, at baseline, 24 h UAER was also measured centrally from a single sample with an immunoturbidimetric method.

#### Follow-up evaluation

In 2015, follow-up data on renal function and cardiovascular events were analysed. Individuals included were followed prospectively as part of the FinnDiane study, but laboratory data were also collected from their medical files. Kidney disease progression was assessed as the fastest eGFR decline quartile and as progression from a lower albuminuria stratum to a higher one. The median number of available serial creatinine measurements was 13 (IQR 7, 23) per individual included; the median proximity from the baseline visit was on average 10.7 years. From those values, eGFR was calculated. Repeated measurement analysis using mixed models was conducted, resulting in smoothed, predicted eGFR values. As the change in eGFR was used as a dependent variable in the next stage of analysis, no covariates were included in the model. Individual time for eGFR measurements was a random effect in the model. For the purpose of the eGFR change, we calculated the percentage annual change assuming linear decline on the log scale by using the first and last predicted eGFR value according to the formula: [(eGFR last/eGFR first) ^ (1/years elapsed between visits) − 1] × 100. Recently, it was shown that the eGFR slope was a relevant surrogate endpoint for renal outcomes in individuals with type 2 diabetes with a broad range of baseline kidney function [16]. Therefore, we defined eGFR decline according to the quartiles of the eGFR slope with the substantial eGFR decline defined as the eGFR slopes in the lowest 25% range, based on a model with eGFR on a log scale [16]. Stable eGFR decline was defined as the middle 50% range of the eGFR decline. In addition, we conducted analyses of sphingomyelin association with other indicators of kidney disease progression, based on the Chronic Renal Insufficiency Cohort (CRIC) study methodology [17] that included individuals with different aetiologies of chronic kidney disease (CKD). Those indicators were: (1) eGFR halving; (2) eGFR <15 ml min^−1^ 1.73 m^−2^; (3) eGFR halving and eGFR <15 ml min^−1^ 1.73 m^−2^; (4) eGFR decrease of >20 ml min^−1^ 1.73 m^−2^; (5) eGFR halving or decrease of >20 ml min^−1^ 1.73 m^−2^; and (6) eGFR decrease of >25% and change of CKD stage.

Similarly, we had serial measurements of UAER during the follow-up and we also collected data from the medical files. The number of follow-up measurements was dependent upon baseline albuminuria status. Progression in albuminuria was defined as the progression from a lower albuminuria stratum to a higher one, with at least two consecutive measurements needed to confirm the stratum change. UAER measurements were performed annually if an individual had normoalbuminuria; however, they were performed every 3 months in those with microalbuminuria. Data on ESRD were retrieved from the Finnish Care Register for Health Care.

Follow-up data on cardiovascular outcomes and death from baseline to the end of 2015 were retrieved from the Finnish Care Register for Health Care and the Cause of Death Register. CHD was defined as patients having had either a myocardial infarction or coronary revascularisation (percutaneous coronary intervention or coronary artery bypass graft) based on ICD-8/9 code 410, ICD-10 codes I21–I22 and the Nordic Classification of Surgical Procedure codes TFN 40, FN1AT, FN1BT, FN1YT, FNF, FNG, FNA, FNB, FNC, FND, FNE and 5311-5315. In addition, data on ischaemic strokes were collected based on ICD-8/9 codes 433 and 434 and ICD-10 code I63.

### Statistical analyses

Analyses were performed using SAS version 9.4 (SAS Institute, Cary, NC, USA) and SPSS Statistics version 21 (IBM, Armonk, NY, USA). Distribution of variables was tested by Kolmogorov–Smirnov test. Continuous variables are given as mean ± SD if normally distributed, and otherwise as median with IQR. Categorical variables are given as percentages. Between-group differences were analysed using ANOVA for normally distributed variables and *χ*^2^ test for categorical variables. Linear association of serum sphingomyelin with clinical variables was assessed using univariable regression; the strength of association is presented with Pearson *r*.

For the multivariable analyses of sphingomyelin association with different renal and CVD outcomes, Cox proportional hazard survival regression or logistic regression analysis or linear regression analysis was used, as appropriate. Variables, measured at baseline visit and reported to be associated with renal disease progression or CHD, were selected into the models together with the sphingomyelin level, including sex, time of diabetes onset, diabetes duration, previous or current smoking, systolic blood pressure, BMI, HbA_1c_, HDL-cholesterol and triacylglycerols. Sex and smoking were modelled as categorical, and other variables as continuous variables, respectively. When used in different models, due to low sphingomyelin level and in the absence of a standard concentration range, sphingomyelin level was scaled per its SD, to give more meaningful association measures. The assumption of the proportional hazards was tested by plotting Schoenfeld residuals against time and testing a nonzero slope by including time–covariate interaction. The potential nonlinearity in the relationship between sphingomyelin and different clinical outcomes was tested using generalised additive modelling (GAM) without a priori assumptions of the shape of the relation. GAM is an extension of the generalised linear model and allows the inclusion of nonparametric smoothing functions to identify a potential nonlinearity in the relationship between the independent and the dependent variables [18]. None of the relationships showed nonlinearity. Assessment of the clinical benefit of using serum sphingomyelin as a predictor of a substantial eGFR decline, alone or on top of the current clinical standard UAER, was performed using receiver operating characteristic (ROC) curves. Finally, a sensitivity analysis was carried out on the effect of sphingomyelin associations by using the approximate Bayesian bootstrap hot-deck imputation method for missing values of eGFR decline. The results were similar and did not change the interpretation. A *p* value of <0.05 was considered statistically significant.

## Results

### Participant characteristics

The participants (*n* = 1087) had a median age of 38.1 (IQR 28.3, 46.9) years, and diabetes duration of 21.9 (IQR 12.8, 31.7) years at baseline. There were more men included in the study (54%).

Serum sphingomyelin concentration was higher in females than in males (0.47 [IQR 0.42, 0.54] vs 0.45 [IQR 0.40, 0.51] absolute concentration units [CU], respectively), and increased with increasing age (*r* = 0.12), diabetes duration (*r* = 0.12) and HbA_1c_ (*r* = 0.18), but decreased with height (*r* = −0.14) (*p* < 0.001 for all the listed associations) (electronic supplementary material [ESM] Table 1). Sphingomyelin was associated with systolic blood pressure (*r* = 0.10, *p* = 0.001) and standard lipid variables, including total cholesterol (*r* = 0.58, *p* < 0.001), HDL-cholesterol (*r* = 0.10, *p* = 0.002) and triacylglycerols (*r* = 0.22, *p* < 0.001). However, sphingomyelin was not associated with lipid-lowering therapy (*r* = 0.03, *p* = 0.3). ESM Table 2 shows clinical characteristics of the study population depending on the quartiles of sphingomyelin level.

### Sphingomyelin and renal outcomes

At baseline, 778 participants had normoalbuminuria, 148 had microalbuminuria and 161 had macroalbuminuria. According to eGFR, 794 participants were classified into the G1 KDIGO eGFR category, 196 into G2 category, 63 into G3 category (32 patients into G3a, 31 patients into G3b), 29 into G4 category and five into G5 category. In the univariable analysis, serum sphingomyelin was associated with eGFR (*r* = −0.11) and UAER (*r* = 0.21), with *p* < 0.001 for both comparisons.

#### Sphingomyelin and the eGFR decline

Table 1 presents baseline characteristics of the studied population according to the rate of the eGFR decline during a median follow-up of 10.7 (IQR 6.4, 13.5) years. Serum sphingomyelin was significantly and directly associated with the lowest quartile of eGFR decline (median yearly eGFR decline −4.4 [IQR −6.8, −3.1] ml min^−1^ 1.73 m^−2^; from −2.4 to −29.6 ml min^−1^ 1.73 m^−2^). The association remained significant even when adjusted for other variables known for their association with GFR decline, including sex, diabetes duration, age of diabetes onset, smoking, blood pressure, HbA_1c_ and BMI (Table 2). Of note, serum sphingomyelin was significantly correlated with eGFR decline after adjustment for lipid variables, such as serum triacylglycerols and HDL-cholesterol. Moreover, the independent association of serum sphingomyelin level with eGFR decline was present independently of the definition of eGFR decline and independently of the baseline eGFR (ESM Tables 3 and 4).

**Table 1:** Baseline clinical characteristics of patients with substantial eGFR decline (the fastest 25% eGFR decline) vs stable change in eGFR (the middle 50% eGFR decline)

Characteristic	Fastest eGFR decline	Stable eGFR decline
*N*	271	544
Sex (M/F, %)	63/37*	54/46
Age at diabetes onset (years)	12.4 (8.6, 18.8)*	14.9 (9.2, 22.6)
Diabetes duration (years)	25.2 (15.0, 34.2)*	20.3 (12.7, 30.3)
Current/ex/never smokers (%)	31/23/45*	28/21/51
BMI (kg/m^2^)	24.5 (22.3, 26.8)*	25.1 (23.0, 27.2)
Waist-to-hip ratio	0.88 ± 0.08	0.87 ± 0.08
Systolic blood pressure (mmHg)	135 (123, 150)*	132 (122, 143)
Diastolic blood pressure (mmHg)	80 (72, 87)	79 (72, 85)
Insulin dose (U/kg)	0.66 (0.53, 0.85)	0.67 (0.52, 0.82)
HbA_1c_ (mmol/mol)	73.8 (62.6, 88.0)*	66.1 (56.3, 77.0)
HbA_1c_ (%)	8.9 (7.9, 10.2)*	8.2 (7.3, 9.2)
eGFR (ml min^−1^ 1.73 m^−2^)	98 (60, 120)*	109 (98, 119)
Normoalbuminuria (%)	48*	76
Microalbuminuria (%)	17*	14
Macroalbuminuria (%)	35*	10
Lipid-lowering medication (%)	17	9
Blood pressure-lowering medication (%)	55*	31
Retinal laser treatment (%)	46*	24
Serum cholesterol (mmol/l)	5.0 (4.4, 5.7)*	4.8 (4.2, 5.4)
HDL-cholesterol (mmol/l)	1.3 (1.0, 1.5)*	1.4 (1.2, 1.6)
Triacylglycerols (mmol/l)	1.2 (0.9, 1.8)*	0.9 (0.7, 1.3)
Serum sphingomyelin (CU)^a^	0.48 (0.42, 0.55)*	0.46 (0.40, 0.52)

Data are presented as mean±SD or as median (25th, 75th percentile), as appropriate

25% fastest eGFR decline had a median yearly eGFR decline −4.4 (IQR −6.8, −3.1) ml min^−1^ 1.73 m^−2^, whereas the stable middle 50% eGFR decline group had a median yearly eGFR decline −0.9 (IQR −1.4, −0.6) ml min^−1^ 1.73 m^−2^

^a^NMR measures are provided in CU based on the trimethylsilyl propanoic acid reference signal

*p < 0.05 for variables with significant difference between the two groups of eGFR decline

**Table 2:** Multivariable binary logistic regression analysis with the fastest 25% eGFR decline as the outcome variable (compared with all other participants or compared with those with stable middle 50% eGFR decline)

Variable	Fastest 25% eGFR decline vs all other		Fastest 25% eGFR decline vs stable 50% eGFR decline	
	OR (95% CI)	*p* value	OR (95% CI)	*p* value
Serum sphingomyelin (1 SD increase in CU)^a^	1.36 (1.15, 1.61)	<0.001	1.27 (1.06, 1.52)	0.010
Sex (male = 1)	1.36 (0.96, 1.92)	0.083	1.25 (0.86, 1.82)	0.236
Diabetes duration (years)	1.02 (1.01, 1.04)	0.006	1.03 (1.01, 1.04)	0.005
Age at diabetes onset (years)	0.99 (0.97, 1.01)	0.28	1.00 (0.97, 1.02)	0.644
Smoking (current vs never)	1.08 (0.74, 1.58)	0.70	1.05 (0.71, 1.57)	0.80
Smoking (ex vs never)	1.08 (0.72, 1.63)	0.71	1.06 (0.68, 1.64)	0.80
Systolic blood pressure (mmHg)	1.01 (1.00, 1.02)	0.034	1.01 (1.00, 1.02)	0.061
HbA_1c_ (mmol/mol)	1.02 (1.01, 1.03)	<0.001	1.02 (1.01, 1.03)	<0.001
BMI (kg/m^2^)	0.91 (0.86, 0.95)	<0.001	0.90 (0.86, 0.95)	<0.001
HDL-cholesterol (mmol/l)	0.30 (0.19, 0.50)	<0.001	0.35 (0.21, 0.61)	<0.001
Triacylglycerols (mmol/l)	1.27 (1.04, 1.54)	0.019	1.28 (1.03, 1.59)	0.024

25% fastest eGFR decline had a median yearly eGFR decline −4.4 (IQR −6.8, −3.1) ml min^−1^ 1.73 m^−2^, whereas the stable 50% eGFR decline group had a median yearly eGFR decline −0.9 (IQR −1.4, −0.6) ml min^−1^ 1.73 m^−2^

^a^NMR measures are provided in CU based on the trimethylsilyl propanoic acid reference signal

#### Sphingomyelin and progression of albuminuria

Also, we tested whether sphingomyelin is associated with albuminuria progression (Table 3). Sphingomyelin was found to be associated with progression from macroalbuminuria to ESRD (*n* = 70 individuals out of 161 with macroalbuminuria; progression rate 57.6 per 1000 person-years), even when adjusted for the whole array of established risk factors, including lipid and glycaemic variables. Higher sphingomyelin values also predicted the occurrence of de novo albuminuria, when adjusted for diabetes duration, time of diabetes onset, sex, smoking, blood pressure and HDL-cholesterol, but not after adjustment for BMI, HbA_1c_ or serum triacylglycerols. Of note, serum sphingomyelin increased the risk of progression from microalbuminuria to macroalbuminuria as well, but the HRs did not reach statistical significance (ESM Table 5).

**Table 3:** Cox regression models showing the HRs for the progression from macroalbuminuria to ESRD (70 individuals progressed to ESRD out of 161 with macroalbuminuria) depending on serum sphingomyelin level

Model	Progression to ESRD	
	HR^a^ (95% CI)	*p* value
Model 1: Serum sphingomyelin	1.53 (1.24, 1.91)	<0.001
Model 2: Model 1 + sex + age of diabetes onset + diabetes duration + smoking	1.56 (1.24, 2.00)	<0.001
Model 3: Model 2 + systolic blood pressure	1.43 (1.14, 1.80)	0.002
Model 4: Model 3 + HDL-cholesterol	1.35 (1.06, 1.72)	0.015
Model 5: Model 4 + triacylglycerols	1.36 (1.06, 1.73)	0.015
Model 6: Model 5 + BMI	1.42 (1.09, 1. 85)	0.009
Model 7: Model 6 + HbA_1c_	1.42 (1.09, 1.86)	0.010
Model 8: Model 3 + BMI + HbA_1c_	1.53 (1.19, 1.97)	0.001

^a^Reported HR increase corresponds to a 1 SD increase in sphingomyelin level

Furthermore, we used GAM to identify a potential nonlinearity in the relationship between the serum sphingomyelin and albuminuria or ESRD. Although visualising suggested that the risk of ESRD started to increase when the sphingomyelin concentration was around 0.50–0.55 CU, nonlinearity was not significant for any of the outcomes, i.e. progression from normoalbuminuria to microalbuminuria (*p* = 0.75), progression from microalbuminuria to macroalbuminuria (*p* = 0.54) or progression from macroalbuminuria to ESRD (*p* = 0.10).

In addition, we analysed whether albuminuria affects the association between serum sphingomyelin and eGFR decline. In the group with the fastest eGFR decline, serum sphingomyelin was higher in those with macroalbuminuria compared with those with normoalbuminuria or microalbuminuria (0.55 ± 0.13 vs 0.47 ± 0.09, *p* < 0.001). Similarly, in the group with the slowest eGFR decline, serum sphingomyelin was significantly higher in the group with macroalbuminuria compared with the group with normoalbuminuria or microalbuminuria (0.48 ± 0.09 vs 0.46 ± 0.09, *p* = 0.02).

Finally, ROC curves were constructed to test whether serum sphingomyelin could add to the prediction of eGFR decline beyond the established risk factor, UAER, at any disease stage (ESM Fig. 1). The analysis showed that serum sphingomyelin level was no better predictor of eGFR decline than albuminuria (AUC 0.588 ± 0.023 vs 0.694 ± 0.023, respectively). Also, adding sphingomyelin to albuminuria (AUC 0.675 ± 0.023) did not result in any improved prediction of eGFR decline compared with albuminuria alone.

### Sphingomyelin and cardiovascular outcomes

During follow-up, 110 CHD events, 60 strokes and 97 deaths occurred. In Cox regression analysis, serum sphingomyelin was significantly and independently associated with incident CHD events, when adjusted for sex, age of diabetes onset, diabetes duration and smoking. However, the association was not significant after adjustment for HbA_1c,_ blood pressure and classical serum lipid variables, including HDL-cholesterol and serum triacylglycerols, albeit increasing the HR in the same direction (Table 4). Serum sphingomyelin was not associated with mortality or stroke (ESM Table 6).

**Table 4:** Cox regression model showing the HRs for incident CHD depending on serum sphingomyelin level (110 individuals suffered an incident CHD event out of 1087 individuals included in the study)

Model	Incident CHD	
	HR^a^ (95% CI)	*p* value
Model 1: Serum sphingomyelin	1.44 (1.19, 1.73)	<0.001
Model 2: Model 1 + sex + age of diabetes onset + diabetes duration + smoking	1.24 (1.01, 1.52)	0.038
Model 3: Model 2 + systolic blood pressure	1.21 (0.98, 1.49)	0.07
Model 4: Model 3 + HDL-cholesterol	1.21 (0.99, 1.48)	0.06
Model 5: Model 4 + triacylglycerols	1.17 (0.96, 1.43)	0.13
Model 6: Model 5 + BMI	1.18 (0.96, 1.44)	0.11
Model 7: Model 6 + HbA_1c_	1.13 (0.92, 1.39)	0.24
Model 8: Model 3 + BMI + HbA_1c_	1.16 (0.94, 1.43)	0.18

The incidence of CHD was 8.2 per 1000 person-years

^a^Reported HR increase corresponds to a 1 SD increase in sphingomyelin level

## Discussion

This study demonstrates a link for serum sphingomyelin with the fastest eGFR decline and with cardiovascular events. Higher serum sphingomyelin is associated with a more rapid eGFR decline, independently of the baseline eGFR and beyond glycaemic control and routinely measured lipid markers. Moreover, we propose that sphingomyelin is implicated in the progression of renal damage at the early as well as the advanced stages, since we observed an association with the appearance of albuminuria as well as with the progression from macroalbuminuria to ESRD. Lastly, we report that higher serum sphingomyelin is associated with an increased risk of CHD in individuals with type 1 diabetes.

The role of sphingolipids in genetic renal disorders has long been recognised. Less is known about the role of sphingomyelin in diabetic nephropathy. The term ‘sphingomyelin’ stands for a family of sphingomyelins, differing in number of carbon atoms attached to the acyl side-chain and each having a distinct role in different cells. Sphingomyelin levels are regulated by de novo synthesis or, alternatively, through dynamic conversions from ceramide by a sphingomyelin synthase or back to ceramide by the sphingomyelinases [19]. In diabetes, it was postulated that, through the increased serum level of sphingomyelinase phosphodiesterase acid-like 3b (SMPDL3b), ceramide accumulates in the podocytes and contributes to podocytopenia [20]. Recently, aberrant sphingolipid metabolism was described also in mesangial cells, possibly contributing to early diabetic nephropathy [21].

In line with preclinical findings, the current study supports the role of sphingomyelin in GFR loss. Moreover, our results suggest sphingomyelin to be involved in incipient and overt nephropathy progression. It is thus possible that sphingomyelin, and even different sphingomyelin subtypes, takes part in different processes at different stages of the disease [22], supported by the observation that its relationship with traditional lipid variables and blood glucose changes by disease stage. We previously reported that patients with progressive albuminuria show a pattern of triacylglycerol enrichment of lipoprotein particles [23]. It may be that these changes are mediated through sphingomyelin since sphingomyelin inhibits the lipolysis of triacylglycerols by interfering with the lipoprotein lipase action [24]. We found only one study [9] that had examined the sphingolipids in relation to nephropathy progression in type 1 diabetes, in 497 patients from the DCCT cohort. However, the DCCT data seem at odds with our findings as well as with previous findings from the cardiovascular field [10, 11, 25]. They demonstrated a protective role of certain, mainly monounsaturated and very long-chain (more than 20 carbon atoms), ceramides regarding the albuminuria progression. Apart from using a different technique for the sphingolipid quantification, which could influence the results, it can be speculated that certain, especially very long carbon chain, ceramide species even confer renoprotection.

We found no additional benefit of adding the sphingomyelin level to albuminuria for the prediction of eGFR deterioration. This may be a result of sphingomyelin acting within the same pathway as the change in albuminuria on kidney function decline. Namely, accumulation of sphingomyelin is implicated in the disorganisation of lipid rafts in the podocytes and may thus contribute to the disruption of the functional glomerular filtration membrane and the albuminuria [26, 27]. Also, there was no statistically significant association between sphingomyelin and the progression from micro- to macroalbuminuria in our study. We do not believe that sphingomyelin is isolated from the processes that affect patients with microalbuminuria, compared with others. Rather, this result may be due to the lack of power in this ‘shifting’ group of patients, especially because the hazard ratios pointed to the same direction of increased risk for albuminuria progression.

Regarding CHD, our results support previous findings in the general population [10, 11]. Higher levels of ceramides, especially long-chain ones with 16–18 carbon atoms, predicted cardiovascular death in patients with stable CHD or acute coronary syndrome [11], and it has been suggested that they play a role in plaque destabilisation [25]. In preclinical studies, sphingolipid enrichment of lipoprotein particles has been shown in atherosclerotic plaques, changing the lipoprotein surface and rendering them more prone to aggregation, oxidation and macrophage attraction [28]. In addition, treatment with the serine palmitoyltransferase inhibitor myriocin reversed endothelial dysfunction and atherosclerosis in streptozotocin-treated [29] and in apolipoprotein E (ApoE)-deficient mice [30]. Notably, most of the studies in humans found a significant association with cardiovascular outcomes only when focused on specific sphingolipid species, and not with the total sphingolipid pool [11, 12]. From our results, where we were able to demonstrate a significant association even between the total sphingomyelin and the coronary events, it is tempting to speculate that in type 1 diabetes the association between sphingomyelin metabolism and cardiovascular outcomes is even stronger.

Several mechanisms might explain the link of sphingomyelin with vascular complications in diabetes. First, sphingomyelin and other metabolites of the sphingolipid pathway have been shown to play a causal role in the development of insulin resistance by inhibition of several intermediates in the insulin signalling pathway, including insulin receptor substrate 1, Akt/PKB and phosphoinositide 3-kinase [31–33], whereby insulin resistance is a well-accepted mechanism promoting both microvascular and macrovascular damage [34, 35]. Second, elevated levels of angiotensin 2 in diabetes activate the rate-limiting enzyme of de novo sphingomyelin synthesis, serine palmitoyltransferase, through angiotensin 2 receptors, increasing ceramide intracellularly, thereby inducing apoptosis and further fuelling the vicious cycle of micro- and macrovascular complications [36]. Third, the sphingomyelin pathway was shown to act downstream of reactive oxygen species (ROS) production [37], mediating adverse effects of an important pathway activated by the hyperglycaemic milieu [38]. Notably, sphingomyelin conversion to ceramide stimulated the ROS production in endothelial cells [39], further aggravating its proinflammatory and proatherogenic role.

Although sphingomyelin was associated with the progression of renal and cardiovascular disease, we cannot conclude whether it is merely a marker of progression or a causal risk factor. Whereas Mendelian randomisation might give insight on the causality, that would require robust genetic risk factors for total serum sphingomyelin. To our knowledge, there are no robust genetic findings for total serum sphingomyelin; further, none of the genetic loci previously reported for sphingomyelin subclasses [40] were directionally and consistently associated with the total serum sphingomyelin in our data (data not shown).

Strengths of this study include the large size of the cohort with the long follow-up, the completeness of data, the reliability of the national registries for the evaluation of cardiovascular outcomes [3] and the standardised assessment of renal disease with both eGFR and albuminuria. Yet, we acknowledge some limitations. Even though serum sphingomyelin showed consistent association with a wide range of kidney function indicators, serum creatinine may be a biased indicator of kidney function in the setting of diabetes and therefore our results should be confirmed also with other filtration markers, such as cystatin C. Metabolomic analysis enabled us to assess the total serum sphingomyelin level, mainly carried in the circulation by LDL-cholesterol [41]. However, sphingomyelins with different fatty acid side chains may play different roles in specific organs [11]. Thus, the determination of specific sphingomyelins could provide information on target sphingomyelins involved in the renal and cardiovascular disease progression. In addition, physical exercise and dietary habits were not analysed in this study but could have an effect on the sphingomyelin level and could modify its association with renal and cardiovascular outcomes [10, 42]. Nevertheless, the strong association demonstrated in our study of total sphingomyelin with the two most important complications that contribute to the excess mortality in type 1 diabetes urges for further research with the aims, first, to identify specific sphingolipids involved in the renal and cardiovascular disease processes; second, to identify whether de novo sphingomyelin synthesis or rather conversion from ceramide is the main driver of increased sphingomyelin; and, third, to search for novel therapeutic modalities that target sphingomyelin metabolism. Interestingly, although in the current study statin therapy was not associated with decreased sphingomyelin level, statins, fenofibrate and proprotein convertase subtillisin/kexin type 9 deficiency have been shown to reduce the levels of certain sphingolipids to some extent [43–45]. Lack of the statin effect of reducing the sphingomyelin level in our study might be due to confounding by indication, since those with higher sphingomyelin could also be those more likely to receive statin therapy.

In conclusion, this study extends previous knowledge on the role of lipids in renal and cardiovascular disease by demonstrating that sphingomyelin is associated with the progression of renal disease and CHD in type 1 diabetes. These results imply that current lipid-lowering strategies may not be effective enough to address all the lipid abnormalities contributing to vascular damage. Therefore, further understanding of the complexity of lipid pathways may lead to new therapies interfering with the abnormal lipid signalling, and possibly reduce the burden of diabetes complications and premature mortality.

## Electronic supplementary material

ESM 1 (PDF 351 kb)

## Data Availability

Data regarding the additional analyses are available by request. The ethical statement and the informed consent do not allow the data to be freely available.

## Abbreviations

CKD: Chronic kidney disease
CU: Absolute concentration unit
DKD: Diabetic kidney disease
ESRD: End-stage renal disease
FinnDiane: Finnish Diabetic Nephropathy
GAM: Generalised additive modelling
ROC: Receiver operating characteristic
ROS: Reactive oxygen species
UAER: Urinary AER
