# Supplementary material for: Sphingomyelin and progression of renal and coronary heart disease in individuals with type 1 diabetes
Source: Diabetologia. 2020 Jun 20;63(9):1847–56. doi: 10.1007/s00125-020-05201-9 (PMC7406485; doi:10.1007/s00125-020-05201-9)
Supplement: Supplementary file 1 — (PDF 351 kb) [file 125_2020_5201_MOESM1_ESM.pdf]

Electronic Supplementary Material (SEM)

ESM Table 1. Bivariate associations between sphingomyelin and baseline variables.

Variable	Pearson’s correlation coefficient	*p* value
Age	0.12	<0.001
Diabetes duration	0.12	<0.001
Age at diabetes onset	-0.02	0.5
BMI	0.07	0.02
Height	-0.14	<0.001
Waist-to-hip ratio	-0.04	0.15
Systolic blood pressure	0.10	0.001
HbA_{1c}	0.18	<0.001
Total cholesterol	0.58	<0.001
HDL-cholesterol	0.10	0.002
Serum triacylglicerols	0.22	<0.001
eGFR	-0.11	0.001
UAER	0.21	<0.001

ESM Table 2. Baseline data of the cohort studied according to the highest and lowest quartile of sphingomyelin concentration.

Characteristic	highest sphingomyelin quartile	lowest sphingomyelin quartile
N	272	271
Sex (M/F, %)	47/53	62/38*
Age at diabetes onset (years)	14.1 (9.0, 21.3)	14.1 (9.2, 22.6)
Diabetes duration (years)	24.2 (15.7, 32.6)	18.7 (9.2, 30.9)*
Current/ex/never smokers (%)	33/25/42	24/21/55*
BMI (kg/m^2)	25.0 (22.7, 27.8)	24.1 (22.5, 26.4)*
Waist-to-hip ratio	0.86 ± 0.08	0.87 ± 0.07
Systolic blood pressure (mmHg)	134 (122, 146)	132 (121, 140)*
Diastolic blood pressure (mmHg)	80 (72, 86)	78 (71, 83)*
Insulin dose (IU/kg)	0.67 (0.54, 0.83)	0.67 (0.50, 0.83)
HbA_{1c} (mmol/mol)	72.7 (60.7, 83.9)	65.0 (55.2, 74.9)*
HbA_{1c} (%)	8.8 (7.7, 9.8)	8.1 (7.2, 9.0)*
eGFR (ml/min/1.73m^2)	98 (75, 112)	109 (96, 120)*
Normoalbuminuria (%)	56	80*
Microalbuminuria (%)	16	13*
Macroalbuminuria (%)	28	7*
Lipid lowering medication (%)	12	14
Blood pressure lowering medication (%)	47	31*
Retinal laser treatment (%)	37	23*
Serum cholesterol (mmol/l)	5.6 (5.0, 6.1)	4.2 (3.8, 4.7)*
HDL-cholesterol (mmol/l)	1.4 (1.1, 1.7)	1.3 (1.1, 1.5)*

Triacylglycerols (mmol/l)	1.2 (0.9, 1.8)	0.9 (0.7, 1.3)*
Serum sphingomyelin (CU)#	0.58 (0.54, 0.63)	0.37 (0.34, 0.39)

#NMR measures are provided in CU that are based on the trimethylsilyl propanoic acid reference signal. Data are presented as mean ± standard deviation or as median (25th, 75th percentile), as appropriate. * stands for *p* value <0.05 for variables with significant difference between the 2 groups of sphingomyelin (SM) level.

ESM Table 3. A. Multivariable linear regression model for annual percentage change in eGFR (ml/min/1.73 m^2/year). Adjusted R^2 of the model 0.183; *p* value <0.001.

Variable	B coefficient	95% CI	β	*p* value
Serum sphingomyelin (1SD increase in CU#)	-0.012	-0.016; -0.008	-0.172	<0.001
Sex (male=1)	-0.002	-0.011; 0.007	-0.014	0.66
Diabetes duration (years)	0.000	-0.001; 0.000	-0.058	0.11
Age at diabetes onset (years)	0.000	0.000; 0.001	0.047	0.16
Smoking (current vs. never)	-0.001	-0.010; 0.009	-0.005	0.88
Smoking (ex vs. never)	-0.005	-0.016; 0.005	-0.029	0.32
Systolic blood pressure (mmHg)	-0.001	-0.001; 0.000	-0.184	<0.001
HbA_{1c} (mmol/mol)	-0.001	-0.001; 0.000	-0.130	<0.001
BMI (kg/m^2)	0.003	0.001; 0.004	0.134	<0.001
HDL-cholesterol (mmol/L)	0.032	0.021; 0.044	0.181	<0.001
Triacylglycerols (mmol/L)	-0.012	-0.017; -0.006	-0.137	<0.001
Constant	0.017	-0.034; 0.069		0.51

#NMR measures are provided in CU that are based on the trimethylsilyl propanoic acid reference signal.
B coefficient - the unstandardised regression coefficient, β - a standardised regression coefficient.

B. Multivariable linear regression model for annual percentage change in eGFR (ml/min/1.73 m^2/year), adjusted for the baseline eGFR. Adjusted R^2 of the model 0.329; *p* value <0.001.

Variable	B coefficient	95% CI	β	*p* value
Serum sphingomyelin (1SD increase in CU#)	-0.008	-0.012; -0.004	-0.116	<0.001
baseline eGFR (ml/min/1.73m^2)	0.001	0.001; 0.001	0.479	<0.001
Sex (male=1)	-0.012	-0.020; -0.004	-0.089	0.002
Diabetes duration (years)	0.001	0.001; 0.001	0.195	<0.001
Age at diabetes onset (years)	0.001	0.001; 0.002	0.155	<0.001
Smoking (current vs. never)	-0.003	-0.012; 0.005	-0.020	0.46
Smoking (ex vs. never)	0.002	-0.008; 0.011	0.009	0.74
Systolic blood pressure (mmHg)	0.000	-0.001; 0.000	-0.116	<0.001
HbA1c (mmol/mol)	-0.001	-0.001; 0.000	-0.149	<0.001
BMI (kg/m^2)	0.002	0.001; 0.004	0.127	<0.001
HDL-cholesterol (mmol/L)	0.024	0.013; 0.034	0.134	<0.001
Triacylglicerols (mmol/L)	-0.008	-0.013; -0.003	-0.091	0.002
Constant	-0,167	-0.219; -0.114		<0.001

#NMR measures are provided in CU that are based on the trimethylsilyl propanoic acid reference signal.
B coefficient - the unstandardised regression coefficient, β - a standardised regression coefficient.

C. Binary logistic regression models for variables associated with the 25% fastest eGFR decline (vs. all others) according to the baseline eGFR below or above 60 or 90 ml/min/1.73m^2.

Variable	eGFR ≥90 ml/min/m^2		60 < eGFR ≤ 90 ml/min/m^2		eGFR <60 ml/min/m^2	
	n=142/794		n=42/196		n=59/97	
	OR (95% CI)	*p* value	OR (95% CI)	*p* value	OR (95% CI)	*p* value
Serum sphingomyelin (1SD increase in CU[#])	1.32 (1.05-1.65)	0.019	0.93 (0.63-1.38)	0.71	1.84 (1.01-3.37)	0.047
Sex (male=1)	1.88 (1.19-2.99)	0.007	1.16 (0.51-2.62)	0.73	0.80 (0.22-2.90)	0.73
Diabetes duration (years)	1.01 (0.99-1.03)	0.42	1.03 (0.99-1.08)	0.13	0.98 (0.92-1.05)	0.60
Age at diabetes onset (years)	0.98 (0.95-1.01)	0.18	1.02 (0.97-1.07)	0.44	0.98 (0.89-1.07)	0.58
Smoking (current vs.never)	1.08 (0.68-1.69)	0.75	1.06 (0.38-2.99)	0.91	2.15 (0.34-13.83)	0.42
Smoking (ex vs. never)	1.02 (0.59-1.78)	0.94	1.00 (0.40-2.50)	0.99	1.18 (0.34-4.12)	0.79
Systolic blood pressure (mmHg)	1.00 (0.99-1.02)	0.95	1.01 (0.99-1.03)	0.45	1.01 (0.98-1.03)	0.58
HbA1c (mmol/mol)	1.02 (1.01-1.03)	0.03	1.03 (1.00-1.06)	0.05	1.04 (1.00-1.08)	0.09
BMI (kg/m^2)	0.91 (0.85-0.97)	0.003	0.92 (0.82-1.04)	0.18	0.98 (0.84-1.14)	0.80
HDL-cholesterol (mmol/L)	0.39 (0.21-0.74)	0.004	0.43 (0.15-1.26)	0.12	0.24 (0.04-1.37)	0.11
Triacylglicerols (mmol/L)	1.35 (1.05-1.73)	0.018	1.45 (0.81-2.60)	0.21	0.61 (0.30-1.21)	0.16

#NMR measures are provided in CU that are based on the trimethylsilyl propanoic acid reference signal.
B coefficient - the unstandardised regression coefficient, β - a standardised regression coefficient.

	eGFR halving		eGFR<15 ml/min/1.73m2		eGFR halving and eGFR<15 ml/min/1.73m2		eGFR decrease of >20 ml/min/1.73m^2		eGFR halving or decrease of >20 ml/min/1.73m2		eGFR decrease of >25% and change of CKD stage	
	n=100		n=87		n=73		n=222		n=234		n=176	
Variable	OR (95% CI)	*p value*	OR (95% CI)	*p* value	OR (95% CI)	*p* value	OR (95% CI)	*p* value	OR (95% CI)	*p* value	OR (95% CI)	*p* value
Serum sphingomyelin (1SD incr. in CU)	1.53 (1.20-1.96)	0.001	1.85 (1.41-2.43)	<0.001	1.67 (1.26-2.20)	<0.001	1.28 (1.08-1.52)	0.005	1.33 (1.12-1.58)	0.001	1.37 (1.13-1.67)	0.002
Sex (male=1)	1.17 (0.69-1.98)	0.56	0.77 (0.43-1.36)	0.37	0.83 (0.45-1.51)	0.54	1.32 (0.93-1.89)	0.12	1.23 (0.87-1.75)	0.24	0.93 (0.62-1.40)	0.72
Diabetes duration (years)	1.05 (1.02-1.07)	<0.001	1.03 (1.00-1.06)	0.028	1.03 (1.00-1.06)	0.027	1.01 (0.99-1.02)	0.31	1.01 (1.00-1.03)	0.15	1.05 (1.03-1.07)	<0.001
Age at diabetes onset (years)	0.98 (0.95-1.01)	0.16	0.98 (0.94-1.01)	0.229	0.98 (0.95-1.02)	0.32	0.98 (0.96-1.01)	0.13	0.98 (0.96-1.00)	0.10	1.00 (0.98-1.03)	0.74
Smoking (curr. vs.never)	1.18 (0.68-2.07)	0.55	0.99 (0.53-1.84)	0.96	0.91 (0.47-1.75)	0.77	1.12 (0.77-1.64)	0.54	1.05 (0.72-1.54)	0.78	1.08 (0.69-1.69)	0.75
Smoking (ex vs. never)	1.25 (0.69-2.26)	0.46	1.41 (0.74-2.66)	0.29	1.40 (0.72-2.71)	0.32	1.00 (0.66-1.53)	0.98	1.10 (0.73-1.65)	0.66	1.37 (0.87-2.18)	0.18
Systolic blood pressure (mmHg	1.02 (1.00-1.03)	0.034	1.03 (1.02-1.05)	<0.001	1.02 (1.01-1.04)	0.003	1.00 (0.99-1.01)	0.69	1.00 (0.99-1.01)	0.59	1.01 (1.00-1.02)	0.043
HbA_{1c} (mmol/mol)	1.04 (1.03-1.06)	<0.001	1.03 (1.01-1.05)	<0.001	1.04 (1.02-1.06)	<0.001	1.02 (1.01-1.03)	<0.001	1.02 (1.01-1.03)	<0.001	1.04 (1.03-1.05)	<0.001
BMI (kg/m^2)	0.93	0.028	0.89	0.002	0.92	0.039	0.97	0.23	0.96	0.10	0.93	0.015

	(0.86-0.99)		(0.82-0.96)		(0.85-1.00)		(0.93-1.02)		(0.92-1.01)		(0.89-0.99)	
HDL-cholesterol (mmol/L)	0.33 (0.16-0.66)	0.002	0.19 (0.08-0.42)	<0.001	0.29 (0.13-0.65)	0.003	0.44 (0.27-0.72)	0.001	0.37 (0.23-0.61)	<0.001	0.30 (0.17-0.53)	<0.001
Triacylglicerols (mmol/L)	1.54 (1.20-1.97)	0.001	1.58 (1.20-2.07)	0.001	1.55 (1.19-2.03)	0.001	1.29 (1.06-1.56)	0.010	1.32 (1.08-1.61)	0.006	1.58 (1.26-1.97)	<0.001

ESM Table 4. Binary logistic regression models showing the association of serum sphingomyelin with eGFR decrease, defined in different ways

ESM Table 5. Cox regression models presenting the HRs for serum sphingomyelin as a risk factor for albuminuria progression.

	Progression to microalbuminuria (*n* = 134/778)		Progression to macroalbuminuria (*n* = 46/148)	
Model	HR* (95% CI)	*p* value	HR* (95% CI)	*p* value
Model 1: Serum sphingomyelin	1.14 (0.95, 1.35)	0.16	2.19 (0.42, 11.41)	0.4
Model 2: Model 1 + sex + age of diabetes onset + diabetes duration + smoking	1.21 (1.01, 1.46)	0.05	2.27 (0.45, 11.41)	0.3
Model 3: Model 2 + systolic blood pressure	1.20 (0.99, 1.45)	0.06	2.28 (0.45, 11.47)	0.3
Model 4: Model 3 + HDL-cholesterol	1.26 (1.04, 1.53)	0.02	2.20 (0.44, 11.05)	0.3
Model 5: Model 4 + triglycerides	1.10 (0.91, 1.33)	0.3	1.15 (0.23, 5.91)	0.9
Model 6: Model 5 + BMI	1.10 (0.91, 1.33)	0.3	1.06 (0.20, 5.61)	0.9
Model 7: Model 6 + HbA_{1c}	0.97 (0.79, 1.19)	0.8	0.50 (0.09, 2.96)	0.4
Model 8: Model 3 + BMI + HbA_{1c}	1.01 (0.82, 1.24)	0.9	1.04 (0.74, 1.46)	0.8

*Reported HR increase corresponds to a one standard deviation (SD) increase in sphingomyelin level.

Progression rate to microalbuminuria – 134 individuals out of 778 with baseline normoalbuminuria, resulting in a progression rate of 20.2 per 1,000 person-years. Progression rate to macroalbuminuria was 46 individuals out of 148 with baseline microalbuminuria, resulting in a progression rate 1.1 per 1,000 person-years.

ESM Table 6. Cox regression models for death and ischemic stroke risk factors in dependence on serum sphingomyelin level.

Model	Variables included	Death (97/1087) HR* (95% CI)	*p* value	Stroke (60/1087) HR* (95% CI)	*p* value
1	Serum sphingomyelin	2.15 (0.82, 5.63)	0.1	1.10 (0.87, 1.39)	0.4
2	1 + gender + age of diabetes onset + diabetes duration + smoking	1.14 (0.40, 3.23)	0.8	1.04 (0.81, 1.32)	0.8

*Reported HR increase corresponds to a one standard deviation (SD) increase in sphingomyelin level. The incidence rate for stroke was 4.3 per 1,000 person-years (60 individuals experienced incident stroke from 1087 included) and mortality rate was 6.7 per 1,000 person-years (97 subjects died from 1087 included in the study).

ESM Fig. 1. ROC curve for serum sphingomyelin and serum sphingomyelin alone or on top of urinary albumin excretion rate (UAER) compared to UAER alone as a predictor of the fastest 25% eGFR decline.

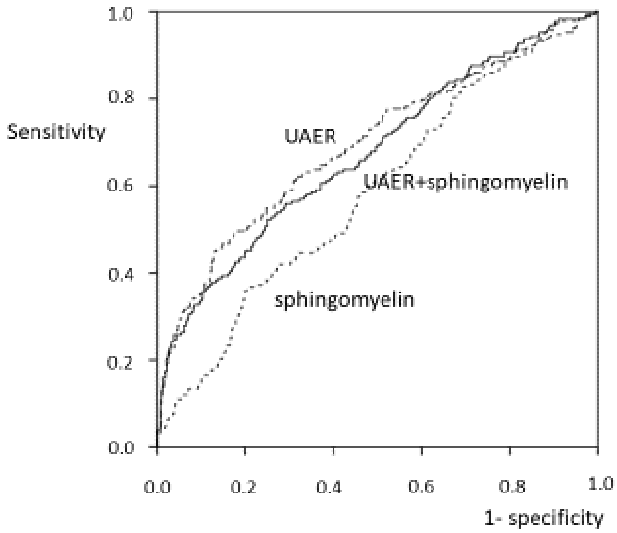

Physicians and nurses at FinnDiane centers participating in patient recruitment and characterization.

The Finnish Diabetic Nephropathy Study Centers

Anjalankoski Health Center	S.Koivula, T.Uggeldahl
Central Finland Central Hospital, Jyväskylä	T.Forslund, A.Halonen, A.Koistinen, P.Koskiaho, M.Laukkanen, J.Saltevo, M.Tiihonen
Central Hospital of Åland Islands, Mariehamn	M.Forsen, H.Granlund, A.-C.Jonsson, B.Nyroos
Central Hospital of Kanta-Häme, Hämeenlinna	P.Kinnunen, A.Orvola, T.Salonen, A.Vähänen
Central Hospital of Kymenlaakso, Kotka	R.Paldanius, M.Riihelä, L.Ryysy
Central Hospital of Länsi-Pohja, Kemi	H.Laukkanen, P.Nyländen, A.Sademies
Central Ostrobothnian Hospital District, Kokkola	S.Anderson, B.Asplund, U.Byskata, P.Liedes, M.Kuusela, T.Virkkala
City of Espoo Health Center:	
Espoonlahti	A.Nikkola, E.Ritola
Tapiola	M.Niska, H.Saarinen
Samaria	E.Oukko-Ruponen, T.Virtanen
Viherlaakso	A.Lyytinen
City of Helsinki Health Center:	
Puistola	H.Kari, T.Simonen
Suutarila	A.Kaprio, J.Kärkkäinen, B.Rantaeskola
Töölö	P.Kääriäinen, J.Haaga, A-L.Pietiläinen
City of Hyvinkää Health Center	S.Klemetti, T.Nyandoto, E.Rontu, S.Satuli-Autere
City of Vantaa Health Center:	
Korso	R.Toivonen, H.Virtanen
Länsimäki	R.Ahonen, M.Ivaska-Suomela, A.Jauhiainen
Martinlaakso	M.Laine, T.Pellonpää, R.Puranen
Myyrmäki	A.Airas, J.Laakso, K.Rautavaara
Rekola	M.Erola, E.Jatkola
Tikkurila	R.Lönnblad, A.Malm, J.Mäkelä, E.Rautamo
Heinola Health Center	P.Hentunen, J.Lagerstam
Helsinki University Central Hospital, Department of Medicine, Division of Nephrology	M.Feodoroff, D.Gordin, O.Heikkilä, K.Hietala, J.Fagerudd, M.Korolainen, L.Kyllönen, J.Kytö, S.Lindh, K.Pettersson-Fernholm, M.Rosengård-Bärlund, A.Sandelin, L.Thorn, J.Tuomikangas, T.Vesisenaho, J.Wadén

Herttoniemi Hospital, Helsinki	V.Sipilä
Hospital of Lounais-Häme, Forssa	T.Kalliomäki, J.Koskelainen, R.Nikkanen, N.Savolainen, H.Sulonen, E.Valtonen
Hyvinkää Hospital	L. Norvio, A.Hämäläinen
Iisalmi Hospital	E.Toivanen
Jokilaakso Hospital, Jämsä	A.Parta, I.Pirttiniemi
Jorvi Hospital, Helsinki University Central Hospital	S.Aranko, S.Ervasti, R.Kauppinen-Mäkelin, A.Kuusisto, T.Leppälä, K.Nikkilä, L.Pekkonen
Jyväskylä Health Center, Kyllö	K.Nuorva, M.Tiihonen
Kainuu Central Hospital, Kajaani	S.Jokelainen, K.Kananen, M.Karjalainen, P.Kemppainen, A-M.Mankinen, A.Reponen M.Sankari
Kerava Health Center	H.Stuckey, P.Suominen
Kirkkonummi Health Center	A.Lappalainen, M.Liimatainen, J.Santaholma
Kivelä Hospital, Helsinki	A.Aimolahti, E.Huovinen
Koskela Hospital, Helsinki	V.Ilkka, M.Lehtimäki
Kotka Health Center	E.Pälikkö-Kontinen, A.Vanhanen
Kouvola Health Center	E.Koskinen, T.Siitonen
Kuopio University Hospital	E.Huttunen, R.Ikäheimo, P.Karhapää, P.Kekäläinen, M.Laakso, T.Lakka, E.Lampainen, L.Moilanen, S. Tanskanen L.Niskanen, U.Tuovinen, I.Vauhkonen, E.Voutilainen
Kuusamo Health Center	T.Kääriäinen, E.Isopoussu
Kuusankoski Hospital	E.Kilkki, I.Koskinen, L.Riihelä
Laakso Hospital, Helsinki	T.Meriläinen, P.Poukka, R.Savolainen, N.Uhlenius
Lahti City Hospital	A.Mäkelä, M.Tanner
Lapland Central Hospital, Rovaniemi	L.Hyvärinen, K.Lampela, S.Pöykkö, T.Rompasaari, S.Severinkangas, T.Tulokas
Lappeenranta Health Center	P. Erola, L.Härkönen, P.Linkola, T.Pekkanen, I.Pulli, E.Repo
Lohja Hospital	T.Granlund, K.Hietanen, M.Porrassalmi, M.Saari, T.Salonen, M.Tiikkainen,
Länsi-Uusimaa Hospital, Tammisaari	I.-M.Jousmaa, J.Rinne
Loimaa Health Center	A.Mäkelä, P.Eloranta
Malmi Hospital, Helsinki	H.Lanki, S.Moilanen, M.Tilly-Kiesi
Mikkeli Central Hospital	A.Gynther, R.Manninen, P.Nironen, M.Salminen, T.Vänttinen
Mänttä Regional Hospital	I.Pirttiniemi, A-M.Hänninen

North Karelian Hospital, Joensuu	U-M.Henttula, P.Kekäläinen, M.Pietarinen, A.Rissanen, M.Voutilainen
Nurmijärvi Health Center	A.Burgos, K.Urtamo
Oulaskangas Hospital, Oulainen	E.Jokelainen, P-L.Jylkkä, E.Kaarlela, J.Vuolaspuro
Oulu Health Center	L.Hiltunen, R.Häkkinen, S.Keinänen-Kiukaanniemi
Oulu University Hospital	R.Ikäheimo
Päijät-Häme Central Hospital	H.Haapamäki, A.Helanterä, S.Hämäläinen, V.Ilvesmäki, H.Miettinen
Palokka Health Center	P.Sopanen, L.Welling
Pieksämäki Hospital	V.Sevtsenko, M.Tamminen
Pietarsaari Hospital	M-L.Holmbäck, B.Isomaa, L.Sarelin
Pori City Hospital	P.Ahonen, P.Merisalo, E.Muurinen, K.Sävelä
Porvoo Hospital	M.Kallio, B.Rask, S.Rämö
Raahe Hospital	A.Holma, M.Honkala, A.Tuomivaara, R.Vainionpää
Rauma Hospital	K.Laine, K.Saarinen, T.Salminen
Riihimäki Hospital	P.Aalto, E.Immonen, L.Juurinen
Salo Hospital	A.Alanko, J.Lapinleimu, P.Rautio, M.Virtanen
Satakunta Central Hospital, Pori	M.Asola, M.Juhola, P.Kunelius, M.-L.Lahdenmäki, P.Pääkkönen, M.Rautavirta
Savonlinna Central Hospital	T.Pulli, P.Sallinen, M.Taskinen, E.Tolvanen, T.Tuominen H.Valtonen, A.Vartia, S-L.Viitanen
Seinäjoki Central Hospital	O.Antila, E.Korpi-Hyövälti, T.Latvala, E.Leijala, T.Leikkari, M.Punkari N.Rantamäki, H.Vähävuori
South Karelia Central Hospital, Lappeenranta	T.Ensala, E.Hussi, R.Härkönen, U.Nyholm, J.Toivanen
Tampere Health Center	A.Vaden, P.Alarotu, E.Kujansuu, H.Kirkkopelto-Jokinen, M.Helin, S.Gummerus, L.Calonius, T.Niskanen, T.Kaitala, T.Vatanen
Tampere University Hospital	P. Hannula, I.Ala-Houhala, R.Kannisto, T.Kuningas, P.Lampinen, M.Määttä, H.Oksala, T.Oksanen, A.Putila, H.Saha, K.Salonen, H.Tauriainen, S.Tulokas
Tiirismaa Health Center, Hollola	T.Kivelä, L.Petlin, L.Savolainen
Turku Health Center	A.Artukka, I.Hämäläinen, L.Lehtinen, E.Pyysalo, H.Virtamo, M.Viinikkala, M.Vähätalo
Turku University Central Hospital	K.Breitholz, R.Eskola, K.Metsärinne, U.Pietilä, P.Saarinen, R.Tuominen, S.Äyräpää

**Vaajakoski Health Center**	**K.Mäkinen, P.Sopanen**
**Valkeakoski Regional Hospital**	**S.Ojanen, E.Valtonen, H.Ylönen, M.Rautiainen, T.Immonen**
**Vammala Regional Hospital**	**I.Isomäki, R.Kroneld, L.Mustaniemi, M.Tapiolinna-Mäkelä**
**Vasa Central Hospital**	**S.Bergkulla, U.Hautamäki, V-A.Myllyniemi, I.Rusk**
